# Recurrent Amaurosis Fugax in a Patient after Stanford Type A Dissection Depending on Blood Pressure and Haemoglobin Level

**DOI:** 10.1155/2012/254204

**Published:** 2012-11-11

**Authors:** L. Tomaschütz, M. Dos Santos, J. Schill, F. Palm, A. Grau

**Affiliations:** Neurologische Klinik, Klinikum der Stadt Ludwigshafen, Bremserstrasse 79, 67063 Ludwigshafen, Germany

## Abstract

*Purpose*. A transient painless monocular visual loss due to a decrease in retinal circulation—also known as “amaurosis fugax”—often precedes acute territorial cerebral ischaemia. The case we present underlines the importance of a comprehensive diagnostic workup in patients with amaurosis fugax. *Case Report*. A 44-year-old man who had suffered from a dissection of the ascending aorta (Stanford Type A) five months ago presented with recurrent monocular vision problems. Episodes with sectional vision loss mainly occurred in combination with low blood pressure levels. Furthermore, the haemoglobin level was chronically low (Hb 9.7 mg/dL), and the patient was by mistake on a simultaneous therapy with phenprocoumon and unfractionated heparin. Carotid artery duplex scanning revealed a high-grade stenosis of the proximal right common carotid artery. MR imaging corroborated hypoperfusion in brain area corresponding to the right MCA. *Conclusion*. Our patient is an example in whom transient retinal ischaemic attacks may originate from haemodynamic reasons.

## 1. Introduction

A transient painless monocular visual loss due to a decrease in retinal circulation—also known as “amaurosis fugax”—often heralds acute territorial cerebral ischaemia [1, 2]. The blood flow can be temporarily reduced in either retinal artery, ophthalmic artery, or ciliary artery, which leads to hypoperfusion, and therefore, ends up causing retinal hypoxia [3]. Normally, a full recovery occurs. In most cases, emboli causing transient retinal artery occlusions or transient ischaemic attacks (TIAs) are assumed to originate from an atherosclerotic internal carotid or ophthalmic artery [4]. The possible pathology in these vessels may be an occlusion, a stenosis, or an irregular ulcerated lesion with or without stenosis [5]. Hence, the main reason is arterio-arterial embolism. Less frequent causes may be vasospasm [6], blood hyperviscosity, or hypercoagulability, which all follow a haemodynamic pathophysiological pattern [7].

Classically, patients report the transient monocular vision loss as a “down- or upcoming curtain” that appears into the field of vision. However, descriptions of fogging, blurring, dimming, or only sectorial vision loss are not uncommon and especially in emergency departments; this possible aetiology of vision problems has to be taken into account [4]. 

The duration of the monocular vision loss ranges from a few seconds up to several hours. As most transient retinal artery occlusions are results of stenosis of the ipsilateral internal carotid artery (ICA), the prior diagnostic tools are to be a carotid artery duplex scanning and a CT or MR imaging to look for previous clinically silent strokes. A cardiovascular workup (ECG, Holter 24-hour monitoring and precordial echocardiography) as well as several laboratory tests including lipid panel, blood glucose level, and a blood cell count complete the diagnostic pathway. The following therapy depends on the underlying aetiology: aspirin in a low dose (100 mg) as a secondary prophylaxis should be taken. In case of a stenosis of a higher grade according to the NASCET criteria [8], the appropriate therapy will be a carotid endarterectomy.

We are presenting a case of rarely found transient retinal ischaemic attacks which were the result of stenosis of the common carotid artery due to a dissection of the aorta that led to the corresponding symptoms in correlation with low blood pressure and low level of haemoglobin.

## 2. Case Report

A 44-year-old man presented with recurrent monocular vision problems. Looking through his right eye, he noticed jagged lines which were followed by a circular vision loss. As an epileptic cause was suspected, the patient was sent to hospital for a 24-hour electroencephalography. Five months ago, the patient had suffered from a dissection of the ascending aorta (Stanford Type A) and had received a conduit implantation with a replacement of the aortic valve. Blood pressure had been adjusted very strictly with target values of about 100 mmHg systolic. Episodes with sectional vision loss mainly occurred in combination with low blood pressure levels. Furthermore, the haemoglobin level was chronically low (Hb 9,7 mg/dL). Due to a planned cholecystectomy, the patient was by mistake on a simultaneous therapy with phenprocoumon and unfractionated heparin. On the day of presentation, no neurologic deficit could be objectified.

**Figure 1:**
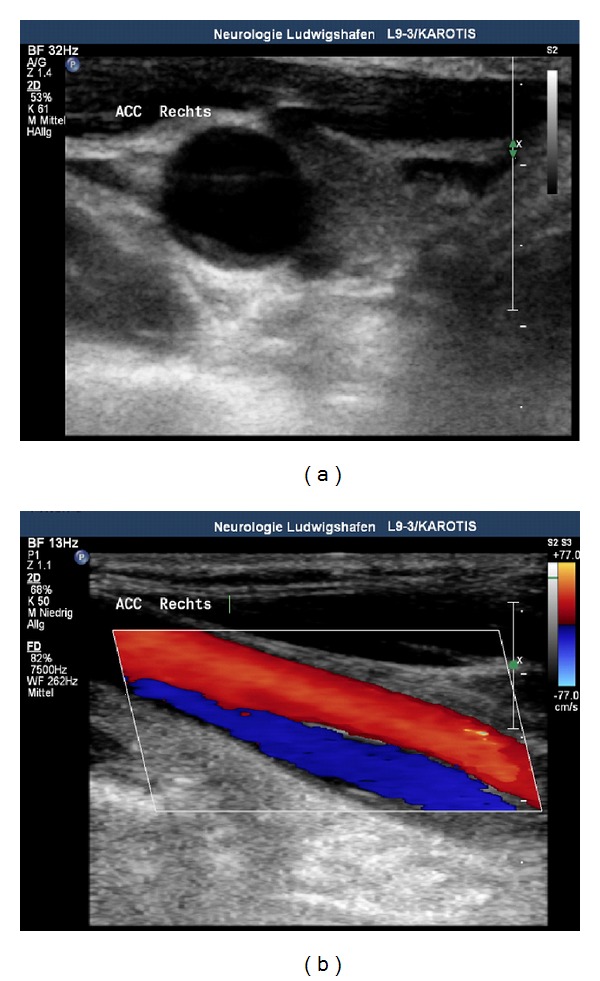
In native scan and with duplex mode, an irregular membrane crossing the vessel with a true and a false lumen was observed.

**Figure 2:**
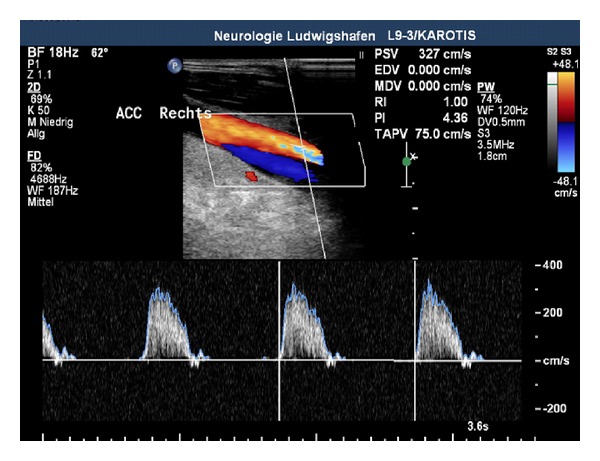
High-grade stenosis of the right common carotid artery (CCA).

**Figure 3:**
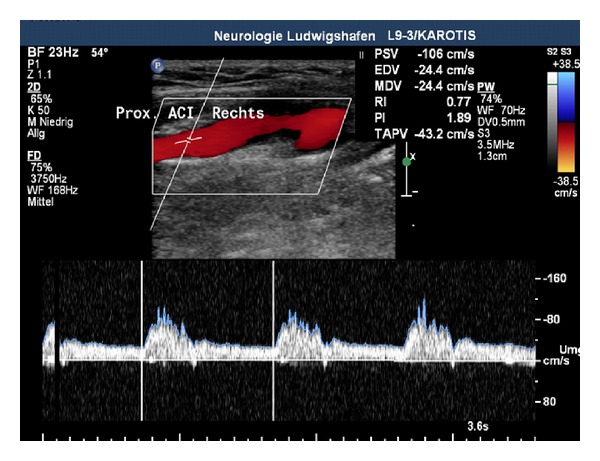
Considerably reduced PSV in the right ICA (automatic detection shows a normal PSV due to the measurement of poststenotic turbulences).

Although the acute incident happened 5 months ago, in carotid artery duplex scanning, an irregular membrane crossing the vessel with a true and a false lumen was still observed (Figure 1). Moreover, a high-grade stenosis of the right common carotid artery with a peak systolic velocity (PSV) of up to 362 cm/sec was revealed (Figure 2). Furthermore, the PSV was considerably reduced in the right ICA as a clear sign of haemodynamic significance (Figure 3). There were no abnormal findings in the posterior circulation.

As a noninvasive tool to estimate the haemodynamic effect of a stenosis of the ICA, a measurement of the cerebrovascular reserve capacity (CVR) using transcranial doppler sonography (TCD) was performed. Arterial carbon dioxide (pCO_2_) is a known variable for cerebral blood flow (CBF) [9], so during breath holding test, an impaired CVR of the right MCA was documented. This was interpreted as a clear sign of haemodynamic relevance [10], and therefore, risk for suffering from a stroke or a TIA was significantly increased [11]. 

**Figure 4:**
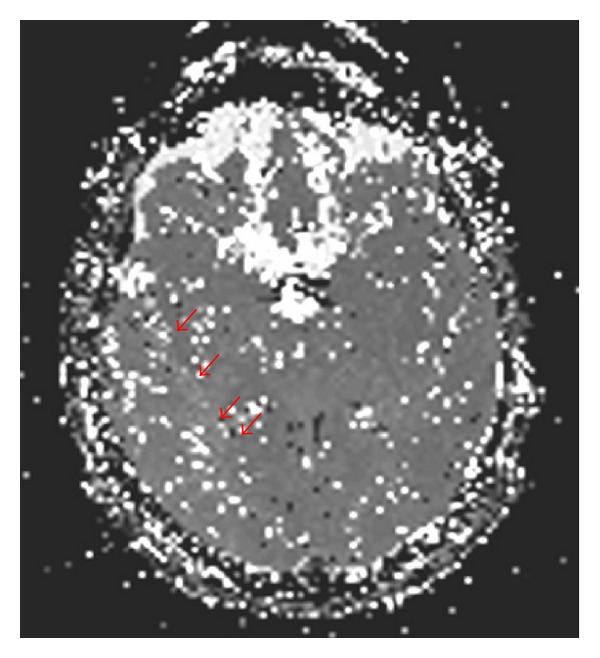
Hypoperfusion in brain area corresponding to the right MCA.

**Figure 5:**
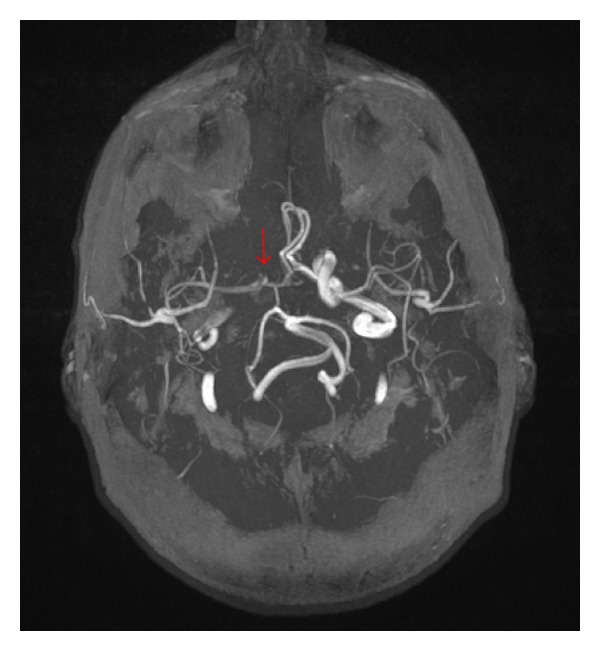
Obvious low flow in right distal ICA.

MR imaging demonstrated hypoperfusion in brain area corresponding to the right MCA (Figure 4) as a sign of haemodynamic impairment in impending watershed infarction [12]. Right distal ICA was clearly less contrasted in MR angiography as an indirect sign of the upstream stenosis of right CCA (Figure 5).

**Figure 6:**
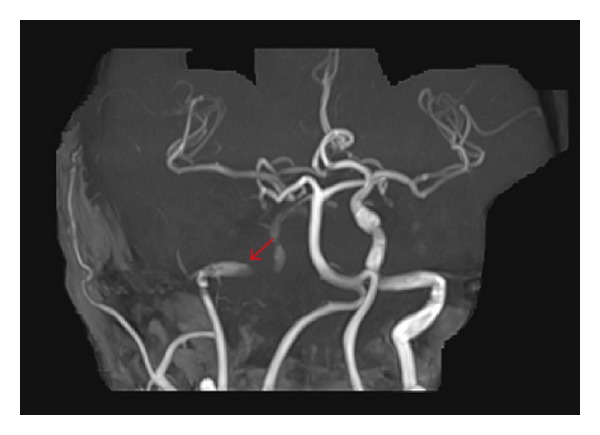
Reduced flow in the right MCA with a cross flow from the left hemisphere.

Moreover, a clearly reduced contrast in the distal part of the right ICA was observed. Finally, the intracranial MR angiography revealed an insufficient cross flow from the left to the right side via the anterior circulation and via ramus communicans posterior resulting in a reduced flow in the right MCA (Figure 6).

**Figure 7:**
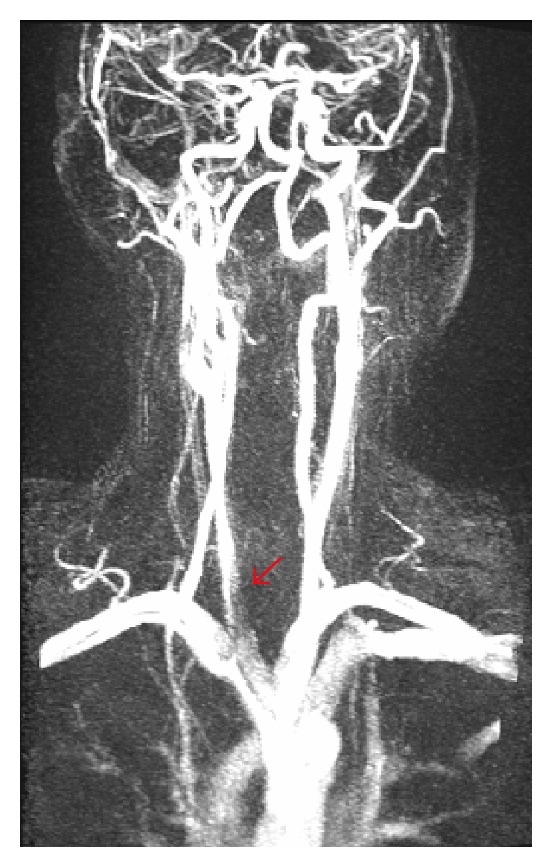
Known dissection Stanford type A with transmitted dissection membrane in brachiocephalic artery, subclavian artery on both sides, and left CCA.

As a result of the dissection, a still existing irregular membrane that narrowed the true lumen was seen in brachiocephalic artery, subclavian artery on both sides and right CCA inducing a functionally relevant stenosis (Figure 7).

## 3. Discussion

Taking all presented findings and the mentioned symptoms into account, we diagnosed our patient with recurrent retinal transient ischaemic attacks. These attacks originated from the combination of very strictly adjusted blood pressure, low haemoglobin levels (due to a misleadingly simultaneous therapy with phenprocoumon and unfractionated heparin), and hypoperfusion induced by CCA stenosis. After reduction of the antihypertensive medication and slowly rising haemoglobin levels, the patient suffered from no further attacks. A decrease in retinal circulation due to a change in posture or an extensive extracranial occlusive disease of the aortic branches is known to cause vascular insufficiency [13].

To draw a conclusion, the case presented underlines the importance of a comprehensive diagnostic workup in patients with amaurosis fugax. Our patient is one of the few examples in whom transient retinal ischaemic attacks may originate from haemodynamic reasons.

## Abbreviations

TIA: Transient ischaemic attack
ICA: Internal carotid artery
CCA: Common carotid artery
PSV: Peak systolic velocity
MCA: Middle cerebral artery
ECD: Extracranial carotid duplex
TCD: Transcranial Doppler sonography
MRA: Magnetic resonance angiogram.
